# Hyperleukocytosis, leukemoid reaction caused by malignant peritoneal mesothelioma: a case report and review of literature

**DOI:** 10.3389/fonc.2025.1550868

**Published:** 2025-05-14

**Authors:** Kaibo Zhu, Dan Zhou, Kang Liu, Lingzhen Wu, Juan Jin, Zimian Luo

**Affiliations:** ^1^ Department of Hematology, Xiangtan Central Hospital, Xiangtan, China; ^2^ The Second Department of Infectious Diseases, Xiangxi Autonomous Prefecture People’s Hospital, Jishou, China

**Keywords:** hyperleukocytosis, malignant peritoneal mesothelioma, paraneoplastic leukemoid reaction, cytokine, glucocorticoids

## Abstract

Paraneoplastic leukemoid reaction (PLR)-induced hyperleukocytosis remains a critical diagnostic challenge. Malignant peritoneal mesothelioma, known for its nonspecific clinical presentation, often evades early detection. We report a 75-year-old male with cirrhosis presenting with fever (38.2°C), progressive ascites, leukocytosis (109.9×10^9^/L), and elevated CRP (247.41 mg/L). Initial diagnosis of spontaneous peritonitis failed to explain the leukocytosis, prompting bone marrow examination, including cytomorphology and genetic analysis, which turned out do not support a diagnosis of leukemia or myeloproliferative neoplasms. Subsequent peritoneal biopsy confirmed diffuse malignant peritoneal mesothelioma. Despite prompt diagnosis, the patient developed progressive multi-organ dysfunction and died on day 14. This case underscores the imperative to consider PLR as a potential cause of hyperleukocytosis in oncological contexts, rather than attributing it solely to hematological malignancies. Moreover, it highlights the importance of considering malignant peritoneal mesothelioma in the differential diagnosis of unexplained ascites and peritoneal thickening. Additionally, we propose the hypothesis that glucocorticoids may hold therapeutic potential in the management of PLR.

## Introduction

Paraneoplastic leukemoid reaction (PLR), an infrequent clinical occurrence, is marked by a significant increase in white blood cell(WBC) counts, often surpassing 50×10^9/L, and is typically correlated with carcinomas of the lung, pancreas, stomach, colorectal region, and renal cell carcinomas (1). Diagnosing PLR is a challenge since leukocytosis is most often associated with bacterial infection (2, 3). However when the leukocyte count exceeds 100×10^9/L, which also known as hyperleukocytosis, there is a high potential for misdiagnosis as a hematological malignancy, such as leukemias or myeloproliferative neoplasms, thereby overshadowing the presence of an underlying solid tumor (4). Malignant peritoneal mesothelioma is an uncommon and highly aggressive form of cancer, with a reported one-year survival rate of around 46% (5). Hyperleukocytosis, when caused by malignant peritoneal mesothelioma, is prone to misdiagnosis or may be overlooked, and is rarely documented in medical literature. In this case report, we present an elderly Chinese male patient exhibiting hyperleukocytosis, which was initially suspected to be spontaneous peritonitis or a hematological malignancy, but was ultimately attributed to a PLR associated with diffuse malignant peritoneal mesothelioma. Our goal is to enhance the understanding of rare conditions among clinical physicians and emphasize the importance of accurate and timely diagnosis to prevent diagnostic delays. The study has been approved by the Medical Ethics Committee (IRB No. 2021-10-002) and informed consent has been obtained from the patients.

## Case report

A 75-year-old Chinese man was admitted to the hospital for worsening fatigue and poor appetite in the last 10 days. He also complained of fever (max temperature was 38.2°C), slight pain in his right lower abdomen, and weight loss of more than 5 kilograms. He had a history of chronic hepatitis B and was diagnosed with liver cirrhosis one year ago. He had never smoked or abused alcohol, and there was no known family history of cancer.

**Figure 1:**
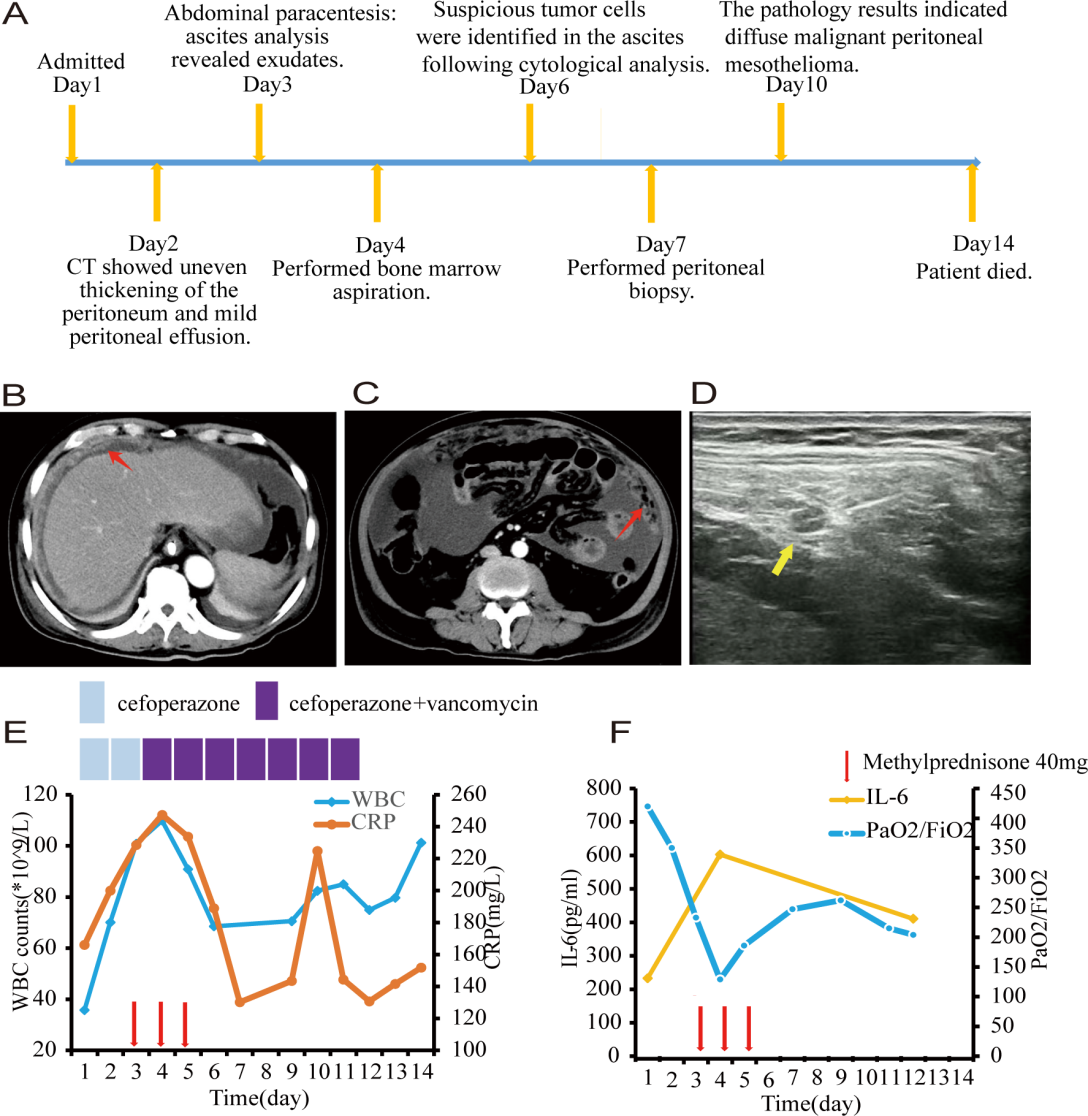
**(A)** Timeline of key clinical events. **(B, C)** Computed tomography scans revealed uneven thickening of the peritoneum, as highlighted by the red arrow. **(D)** Ultrasonographic imaging demonstrated a hypoechoic nodule measuring 1.5 centimeters in diameter with a distinct boundary within the peritoneum, as indicated by the yellow arrow. **(E)** White blood cell counts and C-reactive protein with time. **(F)** Interleukin-6 and PaO2/FiO2 with time. Each red arrow indicates 40 milligram of methylprednisone. Note: PaO2/FiO2: ratio of partial pressure of O2 in arterial blood to fraction of inspired oxygen.

On physical examination, he appeared well with a blood pressure of 116/72 mm Hg. He had mild tenderness in the right lower quadrant without rebound pain. His laboratory blood tests, as detailed in **Table 1**, revealed leukocytosis, with a peripheral blood smear indicating 27% band neutrophils and 60% segmented neutrophils in the absence of blasts. The neutrophil alkaline phosphatase score was 245. Computed tomography showed the liver volume decreased, the spleen was slightly enlarged with uneven thickening of the peritoneum and mild peritoneal effusion (**Figures 1A-C**). Abdominal paracentesis was performed on day 3, and the analysis of his ascites revealed exudates with a gross appearance of yellow. The total nucleated cell count within the fluid was 16×10^9/L, with 60% being polymorphonuclear neutrophils. Biochemical tests of the ascitic fluid indicated a total protein level of 37.2 g/L and a lactate dehydrogenase level of 510 IU/L.

**Table 1:** Laboratory blood test results upon patient admission.

Test item	Test result	Reference range
White Blood Cell Count	35.74	3.9∼9.1 (×10 ^9^/L)
Hemoglobin	135	120∼160 (g/L)
Platelet Count	333	85∼303 (×10 ^9^/L)
C-reactive Protein	166	0∼ 3 (mg/L)
Procalcitonin	5.2	0∼ 0.5 (ng/mL)
Lactic Acid	15.22	0∼ 2 (mmol/L)
Interleukin-6	260	0∼ 5.4 (pg/ml)
Aspartate Aminotransferase	67.8	0∼ 40 (IU/L)
Alanine Aminotransferase	76.1	0∼ 45 (IU/L)
Total Protein Level	73.1	60∼85 (g/L)
Albumin	29.9	35∼55 (g/L)
Lactate Dehydrogenase	423	114∼ 240 (IU/L)
Creatinine	104	53∼115 (umol/L)

Based on the symptoms of fever and abdominal pain, along with leukocytosis and a left shift in the differential count, elevated C-reactive protein(CRP) and procalcitonin as markers of infection, thickening of the peritoneum, and exudative ascites, a diagnosis of post-hepatitis cirrhosis complicated with spontaneous bacteria peritonitis was made and the patient received cefoperazone monotherapy for 48 hours followed by combined with vancomycin to fight against bacterial infection. However, both blood and ascites bacterial cultures were sterile. A metagenomic next-generation sequencing analysis targeting pathogens using a peritoneal fluid sample was performed, which turned out to be negative also. Despite the use of broad-spectrum antibiotics, his symptoms were not alleviated and the WBC count and CRP were getting higher and higher (109.9×10^9^/L and 247.41mg/L, respectively) (**Figure 1E**).

During his leukocytosis, at its peak, the patient experienced respiratory distress. Given concerns regarding leukostasis and potential pulmonary damage from elevated cytokines, he was administered 40mg of methylprednisolone daily for three days. The patient reported significant improvement in his mental state, appetite, and dyspnea, with overall symptom relief. Both the WBC count and CRP levels decreased significantly, however, the improvement was short-lived. Simultaneously, his ascitic fluid volume increased significantly (**Figure 1F**).

**Figure 2:**
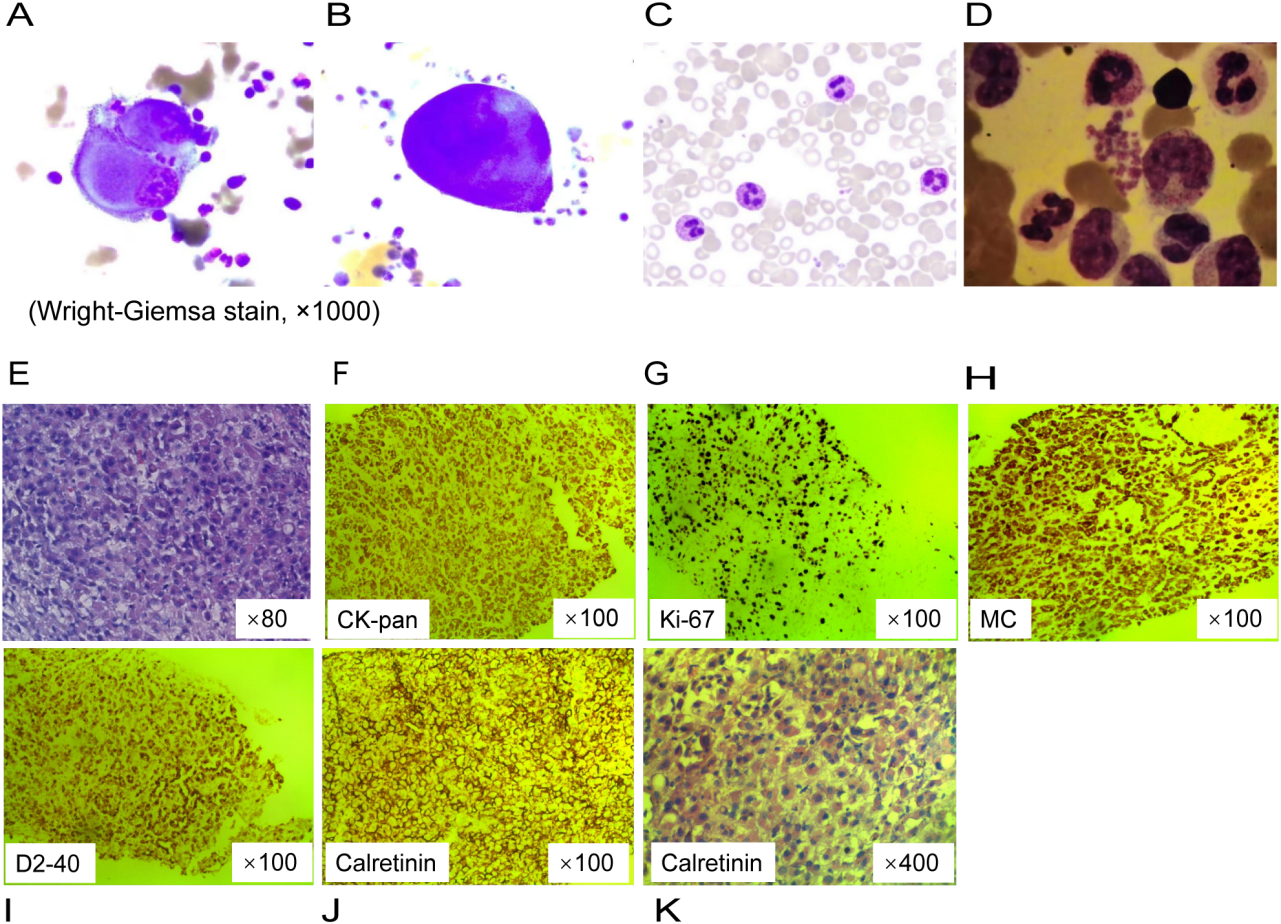
Cytological analysis and pathological findings of peritoneal biopsy tissue. **(A, B)** Clustered giant and abnormally shaped cells with malformed nuclei, and prominant nucleoli on ascites cell smear. **(C)** Segmented neutrophils containing multiple cytoplasmic granules on the peripheral blood smear. **(D)** Bone marrow examination showed left-shifted granulocytic hyperplasia without morphologic dysplasia. **(E)** Diffuse epithelioid cells with pink cytoplasm and nucleolus, focal tumor necrosis on pathology slide of a peritoneal nodule (hematoxylin and eosin stain). **(F–J)** Immunohistochemistry. **(K)** Prominent atypical hyperplastic epithelioid cells, diffusely distributed and arranged, with abundant eosinophilic cytoplasm and displaced nuclei.

Through cytological examinations of the ascites, suspicious tumor cells were identified (**Figures 2A, B**). Subsequently, an ultrasound-guided peritoneal biopsy was performed (**Figure 1D**), and microscopic examination revealed diffuse malignant mesothelioma, the immunohistochemical results showed Vim(+),CK-pan(+),S-100(-),EMA(dim+),CK5/6(+),CR(-),MC6(+),Desmin(focal+),Myogenin(-),Mum-1(-),HMB45(-),CEA(-),D2-40(+),Wt-1(focal,dim+),Villin(-),CDX-2(-), Ki-67(+) (**Figures 2E-K**).

Given the hyperleukocytosis, a bone marrow aspiration was conducted to exclude a hematological malignancy. The morphological analysis revealed a markedly hypercellular marrow without evidence of dysplasia or increased blast cells; the granulocyte-to-erythrocyte ratio was noted to be 4:1 (**Figure 2C, D**). Molecular studies, including polymerase chain reaction for colony stimulating factor 3 receptor (*CSF3R*) mutation and fluorescence *in situ* hybridization for the *BCR::ABL1* fusion gene, were negative, effectively ruling out chronic neutrophilic leukemia and chronic myeloid leukemia, respectively. The bone marrow karyotype was normal at 46, XY [20]. Thus, these findings do not support a diagnosis of leukemia or myeloproliferative neoplasms.

When the patient was first admitted, he believed that his condition was a routine peritonitis and that he would recover quickly after treatment. Unfortunately, he was later diagnosed with a rare malignant neoplasm, and his condition deteriorated day by day. His confidence in treatment was shattered. Ultimately, he chose to receive palliative care. On the 14th day of hospitalization, the patient died of multiple organ failure.

## Discussion

Malignant peritoneal mesothelioma is a rare and highly aggressive disease, accounting for approximately 10-15% of all malignant mesothelioma cases. Its clinical presentation and imaging characteristics can often mimic other intra-abdominal pathologies, such as spontaneous bacterial peritonitis, particularly in patients with liver cirrhosis (6). In this case, the patient presented with leukocytosis and clinical symptoms suggestive of an infection. The cefoperazone administered to the patient is a β-lactam antibiotic, which is primarily used to treat infections caused by susceptible Gram-negative bacteria. Vancomycin, on the other hand, is a broad-spectrum antibiotic that targets resistant Gram-positive bacteria, particularly vancomycin-resistant enterococci. Together, these two antibiotics provide comprehensive coverage against the common bacterial pathogens responsible for peritonitis. However, the lack of improvement in symptoms despite antimicrobial therapy raised the possibility of a different underlying cause. Cytological examination of the ascites and immunohistochemical staining were pivotal in confirming the diagnosis of malignant peritoneal mesothelioma. The positive expression of Vimentin (Vim), Cytokeratin pan (CK-pan), D2-40, and Weighted-100 (Wt-1) provided critical diagnostic evidence. Additionally, bone marrow morphological evaluation, along with clonality assessments through cytogenetic and molecular analyses, ruled out leukemia or myeloproliferative neoplasms. These findings were consistent with the diagnosis of PLR as a response to the diffuse malignant peritoneal mesothelioma.

Literature review has identified 179 cases of PLR, with the esophagus, gallbladder, lung, liver, and pancreas being the most common primary sites. In 22% of cases, the white blood cell count exceeded 100×10^9/L (7). However, the absence of malignant mesothelioma cases in this study suggests that PLR secondary to mesothelioma are infrequently noted and documented. We have summarized 6 cases of malignant mesothelioma as shown in **Table 2** (8–13). In these cases, the longest survival duration was only seven months, suggesting that patients with PLR may have a worse prognosis. Consistent with our findings, a cohort study of 142 pleural mesothelioma cases identified a WBC count exceeding 8.3×10^9/L as a poor prognostic factor (14). Similarly, in other types of tumors, PLR is associated with an aggressive disease course and poor prognosis, and a strong correlation between worsening of tumor burden and aggravation of neutrophilia was observed (15, 16). A cohort study of 17 PLR patients with a WBC count greater than 50×10^9/L reported a very high mortality rate of 76% within the first 30 days, with most patients having disseminated neoplasm (1). In a previous study, clinical outcomes of patients with PLR were poor, with 78%(60/77) patients either discharged to hospice or dying within 12 weeks (17). Patients with a WBC count >100 ×10^9^/L were twice as likely to die as those with the count from 11×10^9^/L to 40 ×10^9^/L (7). Consistent with the reported data, our case rapidly deteriorated and resulted in death within 14 days.

**Table 2:** Cases of malignant mesothelioma with leukemic reaction (WBC > 30× 10^9^/L) previously reported.

Authors	Publication year	Patient age(y)	Patient gender	Patient country	Primary site	WBC counts (× 10^9^/L)	Survival
Ohbayashi H,et al. (8)	1999	61	Male	Japan	pleura	85.1	7 months
Kasuga I, et al. (9)	2001	48	Male	Japan	pleura	64,7	7 months
Kimura N, et al. (10)	2005	70	Male	Japan	peritoneum	46.3	2 weeks
Nishimura M,et al. (11)	2006	59	Male	Japan	pleura	147.0	4 weeks
Thakral B,et al. (12)	2020	58	Female	USA	peritoneum	284	Unreported
Altshuler PC, et al. (13)	2024	35	Female	USA	peritoneum	52	6 months

The pathogenesis of PLR is not well understood, it is a complex and multifactorial process involving the dysregulated release of various cytokines. A retrospective, single-center study observed a strong correlation between the worsening of tumor burden and the aggravation of neutrophilia during PLR in patients with advanced melanoma. In this study, cytokine measurements revealed an increase in proinflammatory cytokines, including interleukin-6(IL-6) and interferon-γ, as well as granulocyte colony-stimulating factor (G-CSF). Additionally, immunohistochemistry confirmed the infiltration of neutrophils into tumor tissue (18). Through cellular experiments, it has been demonstrated that mutations in ras proto-oncogenes may lead to alterations in the expression of various cytokine genes, including G-CSF, granulocyte-macrophage colony-stimulating factor, IL-1, and IL-6, through both transcriptional and post-transcriptional mechanisms (19). Furthermore, G-CSF production has been confirmed through the detection of G-CSF mRNA in the extracts of primary pericardial mesothelioma and elevated serum G-CSF levels in patients (8, 20). The first case of a malignant mesothelioma expressing both G-CSF and its receptor was reported by Motohiro Nishimura (11). However, there is no absolute correlation between white WBC count and serum G-CSF levels. Thakral reported a similar case in which WBC count peaked at 284×10^9/L while serum G-CSF levels remained normal, reflecting the complexity of the PLR mechanism (7, 12). In our case presentation, due to the lack of test reagents, we did not test for G-CSF, but increased serum IL-6 levels were detected (**Figure 1F**), and the therapeutic response to glucocorticoids was notable underscoring the significant role of cytokines in the pathogenesis of PLR. Glucocorticoids effectively alleviate cytokine storm by inhibiting the production of inflammatory cytokines and the cytokine cascade through multiple mechanisms, including the suppression of key pro-inflammatory transcription factors, modulation of immune cell functions, and blockade of cytokine cascades. This observation necessitates further investigation. In addition to the observed elevation in IL-6 levels, the accumulation of additional data supports the potential emerging role of IL-6 as a therapeutic target for the treatment of PLR.

Hyperleukocytosis, defined by a WBC count exceeding 100×10^9/L, represents a medical emergency. In leukemia, high WBC count and leukocyte aggregates result in leukostasis, disseminated intravascular coagulation, and tumor lysis syndrome, which needs emergent cytoreduction therapy with/without leukopheresis (4, 21, 22). However, hyperleukocytosis in PLR is very rarely reported and marginally analyzed in therapeutic strategies. Few experiences advocated emergent leukapheresis or exchange transfusion for hyperleukocytosis accompanied by respiratory distress or cardiovascular instability in children (23–26). It is much less explored whether glucocorticoid may be needed in leukemoid reaction complicating hyperleukocytosis. In our case, we gave the patient methylprednisone to reduce the leukostasis and damage of cytokines to the lungs, which seemed to have an ephemeral effect, this implies anti-inflammatory therapy may play a role in PLR patients with severe inflammatory response, especially before cancer treatment can be started. Nevertheless, this case report has its limitations, primarily due to its retrospective nature and the lack of a large patient cohort for analysis.

In conclusion, this case highlights the rapid progression and early mortality associated with PLR secondary to malignant peritoneal mesothelioma, underscoring that hyperleukocytosis is not necessarily indicative of a hematological malignancy and warrants vigilance for underlying malignancies to prevent delayed diagnosis and treatment. Additionally, histopathological examination is crucial in cases of unexplained ascites and peritoneal thickening.

## Data Availability

The raw data supporting the conclusions of this article will be made available by the authors, without undue reservation.

## References

[1] Portich JP Faulhaber GAM. Leukemoid reaction: A 21st-century cohort study. Int J Lab Hematol. (2020) 42:134–9. doi: 10.1111/ijlh.13127 31765058

[2] Riley LK Rupert J. Evaluation of patients with leukocytosis. Am Family Physician. (2015) 92:1004–11. 26760415

[3] Potasman I Grupper M. Leukemoid reaction: spectrum and prognosis of 173 adult patients. Clin Infect Dis. (2013) 57:e177–e81. doi: 10.1093/cid/cit562 23994818

[4] Ganzel C Becker J Mintz PD Lazarus HM Rowe JM. Hyperleukocytosis, leukostasis and leukapheresis: practice management. Blood Rev. (2012) 26:117–22. doi: 10.1016/j.blre.2012.01.003 22364832

[5] Ullah A Waheed A Khan J Mishra A Tareen B Nama N. Incidence, survival analysis and future perspective of primary peritoneal mesothelioma (PPM): A population-based study from SEER database. Cancers (Basel). (2022) 14:942–52. doi: 10.3390/cancers14040942 PMC8869829 35205689

[6] Chun C-P Song L-X Zhang H-P Guo D-D Xu G-X Li Y. Malignant peritoneal mesothelioma. Am J Med Sci. (2022) 365(1):99–103. doi: 10.1016/j.amjms.2022.07.008 35940275

[7] Abukhiran I Mott SL Bellizzi AM Boukhar SA. Paraneoplastic leukemoid reaction: Case report and review of the literature. Pathol Res Practice. (2021) 217:153295. doi: 10.1016/j.prp.2020.153295 33341546

[8] Ohbayashi H Nosaka H Hirose K Yamase H Yamaki K Ito M. Granulocyte colony stimulating factor-producing diffuse Malignant mesothelioma of pleura. Intern Med. (1999) 38:668–70. doi: 10.2169/internalmedicine.38.668 10440505

[9] Kasuga I Ishizuka S Minemura K Utsumi K Serizawa H Ohyashiki K. Malignant pleural mesothelioma produces functional granulocyte-colony stimulating factor. Chest. (2001) 119:981–3. doi: 10.1378/chest.119.3.981 11243992

[10] Kimura N Ogasawara T Asonuma S Hama H Sawai T Toyota T. Granulocyte-colony stimulating factor- and interleukin 6-producing diffuse deciduoid peritoneal mesothelioma. Mod Pathol. (2005) 18:446–50. doi: 10.1038/modpathol.3800245 15309018

[11] Nishimura M Itoh K Ito K Yanada M Terauchi K Fushiki S. Autocrine growth by granulocyte colony-stimulating factor in Malignant mesothelioma. Ann Thorac Surg. (2006) 82:1904–6. doi: 10.1016/j.athoracsur.2006.02.009 17062276

[12] Thakral B Loghavi S. Marked paraneoplastic leukemoid reaction in a patient with mesothelioma mimicking a myeloid neoplasm. Blood. (2020) 135:457. doi: 10.1182/blood.2019003936 32027751

[13] Altshuler PC Newman AL Garibay JA. Rapid progression of Malignant peritoneal mesothelioma mimicking a postoperative complication in a young woman: A case report. Am J Case Rep. (2024) 25:e942948. doi: 10.12659/AJCR.942948 38803090 PMC11146238

[14] Edwards JG Abrams KR Leverment JN Spyt TJ Waller DA O’Byrne KJ. Prognostic factors for Malignant mesothelioma in 142 patients: validation of CALGB and EORTC prognostic scoring systems. Thorax. (2000) 55:731–5. doi: 10.1136/thorax.55.9.731 PMC1745842 10950889

[15] Heusinger J Czech P Hengstler H Schaumann F Rückert A Schmid F. Paraneoplastic hyperleukocytosis in lung cancer. Innere Med (Heidelberg Germany). (2022) 63:1312–5. doi: 10.1007/s00108-022-01407-8 PMC9510345 36149442

[16] Marouf A Chapuis N Alary AS Burroni B Kosmider O Alifano M. Paraneoplastic hyperleukocytosis mimicking hematologic Malignancy revealing a localized lung cancer. Ann Thorac Surg. (2020) 109:e203–e6. doi: 10.1016/j.athoracsur.2019.06.064 31408646

[17] Granger JM Kontoyiannis DP. Etiology and outcome of extreme leukocytosis in 758 nonhematologic cancer patients: a retrospective, single-institution study. Cancer. (2009) 115:3919–23. doi: 10.1002/cncr.v115:17 19551882

[18] Zhang X-W Wald A Salzmann M Halama N Hassel JC. Cytokine alterations during paraneoplastic neutrophilia and leukemoid reaction in patients with advanced melanoma. Cancer Immunol Immunother: CII. (2022) 72:509–13. doi: 10.1007/s00262-022-03249-7 PMC9870824 35841421

[19] Demetri GD Ernst TJ Pratt ES Zenzie BW Rheinwald JG Griffin JD. Expression of ras oncogenes in cultured human cells alters the transcriptional and posttranscriptional regulation of cytokine genes. J Clin Invest. (1990) 86:1261–9. doi: 10.1172/JCI114833 PMC296857 2212010

[20] Horio H Nomori H Morinaga S Kikuchi T Tomonari H Kuriyama S. Granulocyte colony-stimulating factor-producing primary pericardial mesothelioma. Hum Pathol. (1999) 30:718–20. doi: 10.1016/S0046-8177(99)90100-4 10374783

[21] Jain R Bansal D Marwaha RK. Hyperleukocytosis: emergency management. Indian J Pediatr. (2013) 80:144–8. doi: 10.1007/s12098-012-0917-3 23180411

[22] Bewersdorf JP Zeidan AM. Hyperleukocytosis and leukostasis in acute myeloid leukemia: can a better understanding of the underlying molecular pathophysiology lead to novel treatments? Cells. (2020) 9:2310–29. doi: 10.3390/cells9102310 PMC7603052 33080779

[23] Haase R Merkel N Diwan O Elsner K Kramm CM. Leukapheresis and exchange transfusion in children with acute leukemia and hyperleukocytosis. A single center experience. Klin Padiatr. (2009) 221:374–8. doi: 10.1055/s-0029-1239533 19890790

[24] Romano MJ Weber MD Weisse ME Siu BL. Pertussis pneumonia, hypoxemia, hyperleukocytosis, and pulmonary hypertension: improvement in oxygenation after a double volume exchange transfusion. Pediatrics. (2004) 114:e264–e6. doi: 10.1542/peds.114.2.e264 15286267

[25] Donoso AF Cruces PI Camacho JF León JA Kong JA. Exchange transfusion to reverse severe pertussis-induced cardiogenic shock. Pediatr Infect Dis J. (2006) 25:846–8. doi: 10.1097/01.inf.0000232630.70138.a2 16940848

[26] Underwood MA Wartell AE Borghese RA. Hyperleukocytosis in a premature infant with intrauterine herpes simplex encephalitis. J Perinatol: Off J California Perinatal Assoc. (2012) 32:469–72. doi: 10.1038/jp.2011.138 22643292

